# The bidirectional association of C-peptide with cardiovascular risk in nondiabetic adults and patients with newly diagnosed type 2 diabetes mellitus: a retrospective cohort study

**DOI:** 10.1186/s12933-022-01636-z

**Published:** 2022-10-03

**Authors:** Shuang-Tong Yan, Jing Sun, Zhao-Yan Gu, Xin-Yu Miao, Li-Chao Ma, Ban-Ruo Sun, Xiao-Min Fu, Hong-Zhou Liu, Guang Yang, Fu-Sheng Fang, Hong Li

**Affiliations:** 1 Department of Geriatric Endocrinology, The Second Medical Center and National Clinical Research Center for Geriatric Diseases, Chinese PLA General Hospital, Beijing, China; 2 Health Management Institute, The Second Medical Center and National Clinical Research Center for Geriatric Diseases, Chinese PLA General Hospital, 28 Fuxing Road, Beijing, 100853 China; 3 Department of Endocrinology, The First Medical Center, Chinese PLA General Hospital, Beijing, China; 4 Department of Geriatric Nephrology, The Second Medical Center and National Clinical Research Center for Geriatric Diseases, Chinese PLA General Hospital, Beijing, China; 5 Department of Health Care, The Second Medical Center and National Clinical Research Center for Geriatric Diseases, Chinese PLA General Hospital, 28 Fuxing Road, Beijing, 100853 China

**Keywords:** C-peptide, Cardiac troponin T, C-reactive protein, Biomarkers, Cardiovascular events, Diabetes

## Abstract

**Background:**

Recent literature reported the biological role of C-peptide, but this role is still controversial and unclear. The primary aim of this study was to investigate associations between C-peptide and cardiovascular biomarkers as well as events.

**Methods:**

A total of 55636 participants who had a health examination from 2017 to 2021 were included. Of them, 6727 participants visited the hospital at least twice. Cardiovascular biomarkers like high-sensitivity C-reactive protein (hs-CRP) and high-sensitivity cardiac troponin T (hs-cTnT) were measured and their relationships with fasting C-peptide were evaluated for all participants. Cardiovascular events were obtained during the last visit and their associations with C-peptide were evaluated for those participants who visited the hospital at least twice.

**Results:**

Among the included participants, 11.1% had a previous type 2 diabetes mellitus (T2DM). In the participants without previous T2DM, the relationships between fasting C-peptide and hs-CRP and hs-cTnT were negative if the value of fasting C-peptide was < 1.4 ng/mL and positive if the value was ≥ 1.4 ng/mL. These relationships remained significant after adjusting for hemoglobin A1c, insulin resistance index, and its interaction with C-peptide, even if the participants were stratified by glucose metabolism status or levels of insulin resistance index. Hazard ratios of cardiovascular events were first decreased and then increased with the increasing of baseline C-peptide levels, though these associations became unsignificant using the multivariate Cox regression model. Unlike the participants without previous T2DM, the associations of C-peptide with cardiovascular biomarkers and events were not significant in the patients with previous T2DM.

**Conclusions:**

The associations of C-peptide with cardiovascular biomarkers and events were different between the participants without previous T2DM and those with previous T2DM. The effect of C-peptide on cardiovascular risk may be bidirectional, play a benefit role at a low level, and play a harmful role at a high level in the nondiabetic adults and the patients with newly diagnosed T2DM.

**Supplementary Information:**

The online version contains supplementary material available at 10.1186/s12933-022-01636-z.

## Introduction

C-peptide and insulin are produced from the same precursor and secreted into circulation in equimolar amounts. Unlike insulin, C-peptide is subjected to negligible hepatic first pass metabolism, has almost a five to ten times longer half-life, and has been recognized as a surrogate marker of pancreatic beta cell function [1–3].

Current research reveals that C-peptide is a biologically active peptide. It has been shown to alleviate hyperglycemia-induced inflammation and has a protective effect against diabetic complications in diabetes mellitus (DM) [4, 5]. C-peptide is also viewed as a therapeutic strategy for preventing diabetic vasculopathy in Type 1 DM (T1DM) [2, 6]. However, recent studies have shown that C-peptide levels appear to positively correlate with arterial stiffness and cardiovascular and overall death in nondiabetic adults [7–10]. Additionally, increased levels of C-peptide might play a causal role in early atherogenesis in patients with Type 2 DM (T2DM) [11, 12]. Overall, the beneficial effects of C-peptide seem to be inconsequential and negative in healthy individuals and in T2DM patients.

Given the limited number of studies examining the effects of C-peptide and the potential for confounding like insulin levels, more studies are necessary to confirm the role of C-peptide, especially in nondiabetic adults and T2DM patients. The purpose of our study was to examine the association between levels of fasting C-peptide, cardiovascular biomarkers, and cardiovascular events in healthy individuals and T2DM patients. We also examined associations stratified by glucose metabolism status and insulin resistance index.

## Methods

### Study participants

**Fig. 1:**
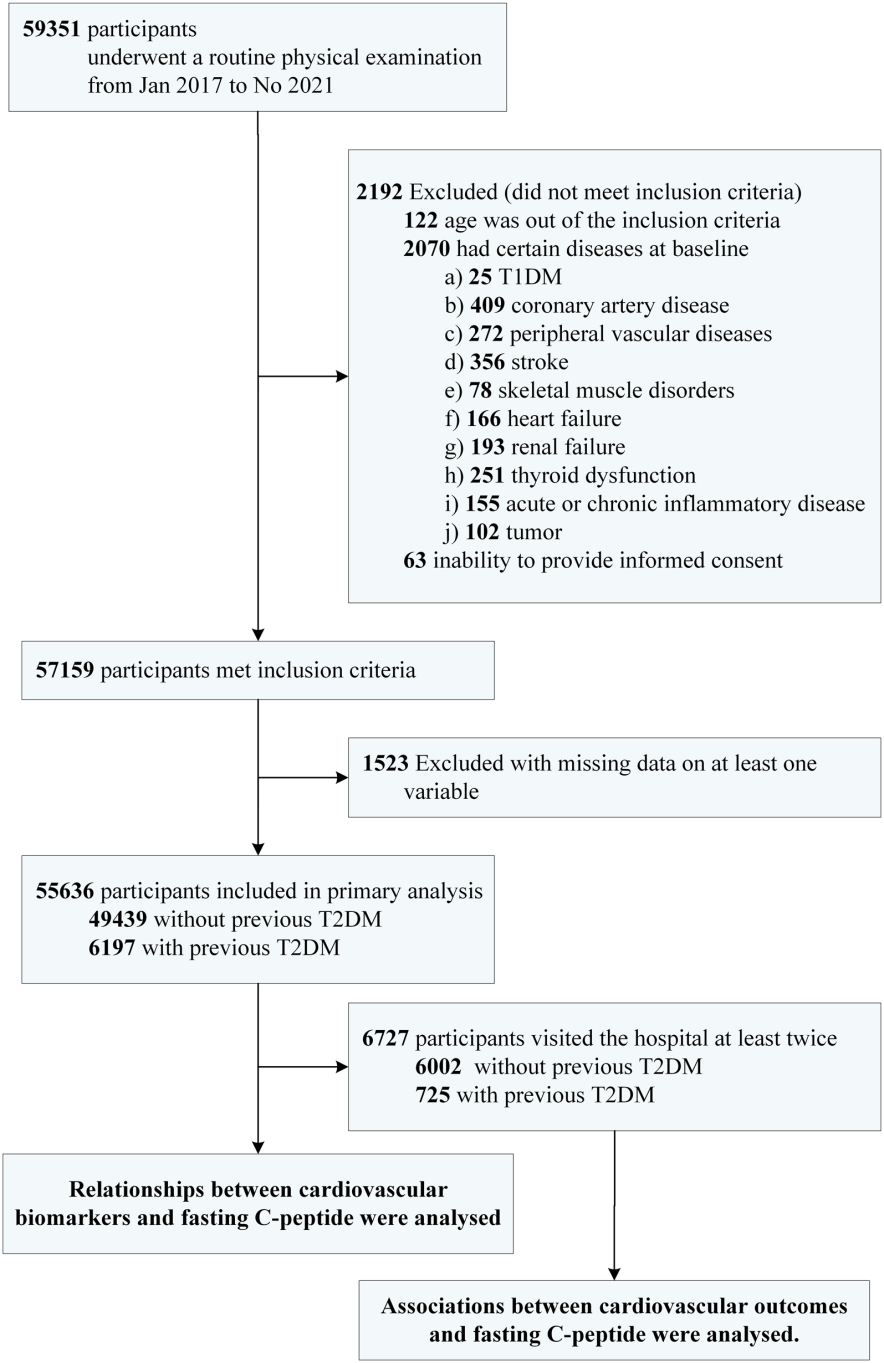
Flow chart of the study population. *T1DM* type 1 diabetes mellitus, *T2DM* type 2 diabetes mellitus

This study was a retrospective cohort study with two stages. First, the study recruited individuals who visited the Chinese PLA General Hospital from Jan 2017 to Nov 2021. The individuals who had undergone a routine physical examination, an oral glucose tolerance test (OGTT), and cardiovascular biomarker measurements during this period were eligible for the participation (n = 59351). Participants with age < 18 years old or > 80 years old, diagnosed diseases of T1DM, coronary artery disease, peripheral vascular diseases, stroke, skeletal muscle disorders, heart failure, renal failure, thyroid dysfunction, acute or chronic inflammatory disease, or tumor, or inability to provide informed consent were excluded (n = 2192). We also excluded 1523 participants with missing data on at least one variable. In the first stage, the study population included 55636 participants. Second, among the included participants in the first stage, 6727 participants visited the hospital at least twice during the study period. The time interval between two visits was no less than six months. Medical information during the last visit was collected for the participants in the second stage. Details for the enrollment were described in Fig. 1.

The study protocol conformed to the Declaration of Helsinki and was approved by the Medical Ethics Committee of the Chinese PLA General Hospital. Written informed consent was given from all participants.

### Data collection and covariates

Information on smoking status were performed via face-to-face interviews. History of diagnosed diseases and medication use were obtained from hospital medical records and confirmed by a medical record review. Previous T2DM was defined as having T2DM diagnosed previously by a clinician and taking antidiabetic medication. Similarly, previous hypertension was defined as having hypertension diagnosed previously by a clinician and taking antihypertensive medication.

Body weight was measured to the nearest 0.1 kg and height to the nearest 0.1 cm. Body mass index (BMI) was calculated as weight in kilograms divided by the square of height in meters. Seated systolic blood pressure (SBP) was measured using an automatic electronic device (HBP-1300, OMRON, Kyoto, Japan). Fasting triglycerides and low-density lipoprotein (LDL) cholesterol were measured directly by routine laboratory methods.

### OGTT and glucose metabolism status

Blood samples were collected in the morning after at least 10 h of overnight fasting. After a 75-g oral glucose load, 2 h postload blood samples were collected. Fasting and 2 h postload plasma glucose levels, were measured by routine laboratory methods using a Hitachi Automatic Analyzer 7600 (Hitachi, Tokyo, Japan), as previously described [13, 14]. Fasting C-peptide levels were measured by chemiluminescence immunoassay, with the limit of detection of 0.01 ng/mL. Fasting insulin levels were measured with the Pharmacia insulin radioimmunoassay kit (Pharmacia Diagnostics, Sweden), with the limit of detection of 0.2 mU/L. Hemoglobin A1c (HbA1c) was measured using high performance liquid chromatography (Variant II, Bio-Rad, Hercules, CA, USA), which was certified by the National Glycohemoglobin Standardization Program.

Glucose metabolism status was assessed by 2 h OGTT and classified into normal glucose metabolism (NGM), prediabetes, and newly diagnosed T2DM (ND-T2DM), in accordance with the 1999 World Health Organization diagnostic criteria [15]. NGM was defined as a fasting plasma glucose (FPG) < 6.1 mmol/L and a 2 h plasma glucose (2hPG) < 7.8 mmol/L. Prediabetes was defined as either an impaired fasting glucose (FPG between 6.1 and 6.9 mmol/L and 2hPG < 7.8 mmol/L) and/or an impaired glucose tolerance (FPG < 7.0 mmol/L and 2hPG between 7.8 and 11.0 mmol/L). ND-T2DM was defined as a FPG ≥ 7 mmol/L and/or a 2hPG ≥ 11.1 mmol/L.

The homeostasis model assessment of insulin resistance (HOMA-IR) was calculated as the product of fasting insulin (in mU/L) and fasting glucose (in mmol/L) levels divided by 22.5.

### Cardiovascular biomarkers and events

Cardiovascular biomarkers included high-sensitivity C-reactive protein (hs-CRP) and high-sensitivity cardiac troponin T (hs-cTnT). Levels of hs-CRP were measured using an immunoturbidimetric assay (Siemens Healthcare Diagnostics, IN, USA), with the limit of detection of 0.01 mg/L. Levels of hs-cTnT were measured by a newly developed high-sensitivity assay on an Elecsys 2010 analyzer (Roche Diagnostics, Indianapolis, Indiana). The limit of detection is 1 ng/L. All the measurements were performed by trained investigators blinded to participant identity and characteristics.

Cardiovascular events included myocardial infarction, ischemic stroke, and hospitalization for unstable angina. All events were obtained from hospital medical records during the last visit for those who visited the hospital at least two times. The diagnosis of cardiovascular events was described previously [16]. The diagnosis of myocardial infarction was based on symptoms, elevated cardiac marker of myocardial necrosis, and the presence of absence of diagnostic electrocardiogram changes [17]. Ischemic stroke was diagnosed according to World Health Organization criteria [18]. Unstable angina was diagnosed using Braunwald’s criteria [19] in addition to documentation of significant coronary artery disease based on coronary angiography. These events were adjudicated by an event adjudication committee who were blinded to the measurements.

### Statistical analysis

Data are shown as mean ± standard deviation (SD) or number (percentage). Variables with nonnormal distribution were log-transformed before parametric testing. First, a locally weighted scatterplot smoothing (LOESS) was drew to assess relationships between serum C-peptide levels and levels of cardiovascular biomarkers in all participants. An optimal cutoff value of fasting C-peptide was obtained from the LOESS analyses. Participants without previous T2DM were grouped by this cutoff value and several multivariate linear regression models were conducted to assess the relationship in each group. All variables except sex and smoking status were standardized before being included in regression models. Associations stratified by glucose metabolism status and HOMA-IR levels were finally performed in sensitivity analyses.

Second, prevalence of cardiovascular events among fasting C-peptide subgroups was calculated for those who visited to the hospital at least twice. Kaplan–Meier survival curve, multivariate Cox proportional hazards model, and restricted cubic spline were performed to evaluate associations between fasting C-peptide and cardiovascular events. Hazard ratio (HR) and 95% confidence interval (CI) were shown.

All analyses were conducted by using R version 4.1.2 (R Foundation for Statistical Computing, Vienna, Austria). A two-sided P value < 0.05 was considered as statistically significant.

## Results

### Demographic characteristics of the participants

A total of 55636 participants were finally included in the first stage of the study. Among them, 11.1% (n = 6197) had previous T2DM. As shown in Table 1, those with previous T2DM had higher levels of fasting C-peptide, HOMA-IR, hs-CRP, and hs-cTnT compared to their counterparts without previous T2DM (all P < 0.0001). The proportion of NGM, prediabetes, and ND-T2DM were 51.5%, 37.3%, and 11.2%, respectively in the participants without previous T2DM. More than seven in ten patients with previous T2DM (71.2%) took metformin and nearly three in ten (27.3%) took insulins.

**Table 1:** Demographic characteristics of the participants

	Participants without previous T2DM	Patients with previous T2DM
Number	49439	6197
Age, year	50.0 ± 9.80	55.9 ± 8.97
Sex		
Men	32539 (65.8)	5182 (83.6)
Women	16900 (34.2)	1015 (16.4)
Current smoking	10238 (20.7)	1752 (28.3)
Previous hypertension		
No	38170 (77.2)	2905 (46.9)
Yes	11269 (22.8)	3292 (53.1)
Lipid-lowering medication use		
No	42758 (86.5)	4185 (67.5)
Yes	6681 (13.5)	2012 (32.5)
BMI, kg/m^2^	25.0 ± 3.48	26.3 ± 3.35
SBP, mmHg	121 ± 18.5	129 ± 18.5
LDL cholesterol, mmol/L	3.17 ± 0.88	2.87 ± 0.98
Triglycerides, mmol/L	1.80 ± 1.46	2.20 ± 1.95
FPG, mmol/L	5.46 ± 1.03	7.76 ± 2.35
Fasting insulin, mU/L	10.7 ± 6.96	13.4 ± 23.8
Fasting C-peptide, ng/mL	2.51 ± 0.97	2.68 ± 1.14
2hPG, mmol/L	8.09 ± 2.39	11.7 ± 3.47
HbA1c, %	5.7 ± 0.63	7.1 ± 1.26
HOMA-IR	2.69 ± 2.13	4.71 ± 8.60
hs-CRP, mg/L	1.69 ± 4.26	2.03 ± 5.23
hs-cTnT, ng/L	5.85 ± 4.82	8.90 ± 27.0
Glucose metabolism status		
NGM	25463 (51.5)	
Prediabetes	18451 (37.3)	
ND-T2DM	5525 (11.2)	
Use of antidiabetic medication		
Metformin		4415 (71.2)
Sulfonylurea		709 (11.4)
Glinides		323 (5.21)
Thiazolidinediones		149 (2.40)
α-Glucosidase inhibitor		1093 (17.6)
DPP-4 inhibitors		1275 (20.6)
GLP-1 agonists		241 (3.89)
SGLT2 inhibitors		401 (6.47)
Insulins		1689 (27.3)

*BMI* body mass index, *DDD-4* dipeptidyl peptidase-4, *FPG* fasting plasma glucose, *GLP-*1 glucagon-like peptide-1, *HbA1c* hemoglobin A1c, *HOMA-IR* homeostasis model assessment of insulin resistance, *hs-CRP* high sensitivity C-reactive protein, *hs-cTnT* high-sensitivity cardiac troponin T, *LDL cholesterol*, low-density lipoprotein cholesterol, *ND-T2DM* newly diagnosed T2DM, *NGM* normal glucose metabolism, *SBP* systolic blood pressure, *SGLT2* sodium-glucose cotransporter 2, *T2DM* type 2 diabetes mellitus, *2hPG* 2 h plasma glucose

In the second stage of the study, 6727 participants who visited the hospital at least twice were included. The median (the interquartile) of the time interval between the first visit and the last visit was 714 days (402 days, 1047 days). Baseline characteristics and the occurrence of cardiovascular events are shown in Additional file 1: Table S1. A total of 300 participants (5.0%) in those without previous T2DM and 98 participants (13.5%) in the patients with previous T2DM had got cardiovascular events during the visits.

### LOESS analyses of serum C-peptide levels and cardiovascular biomarker levels

**Fig. 2:**
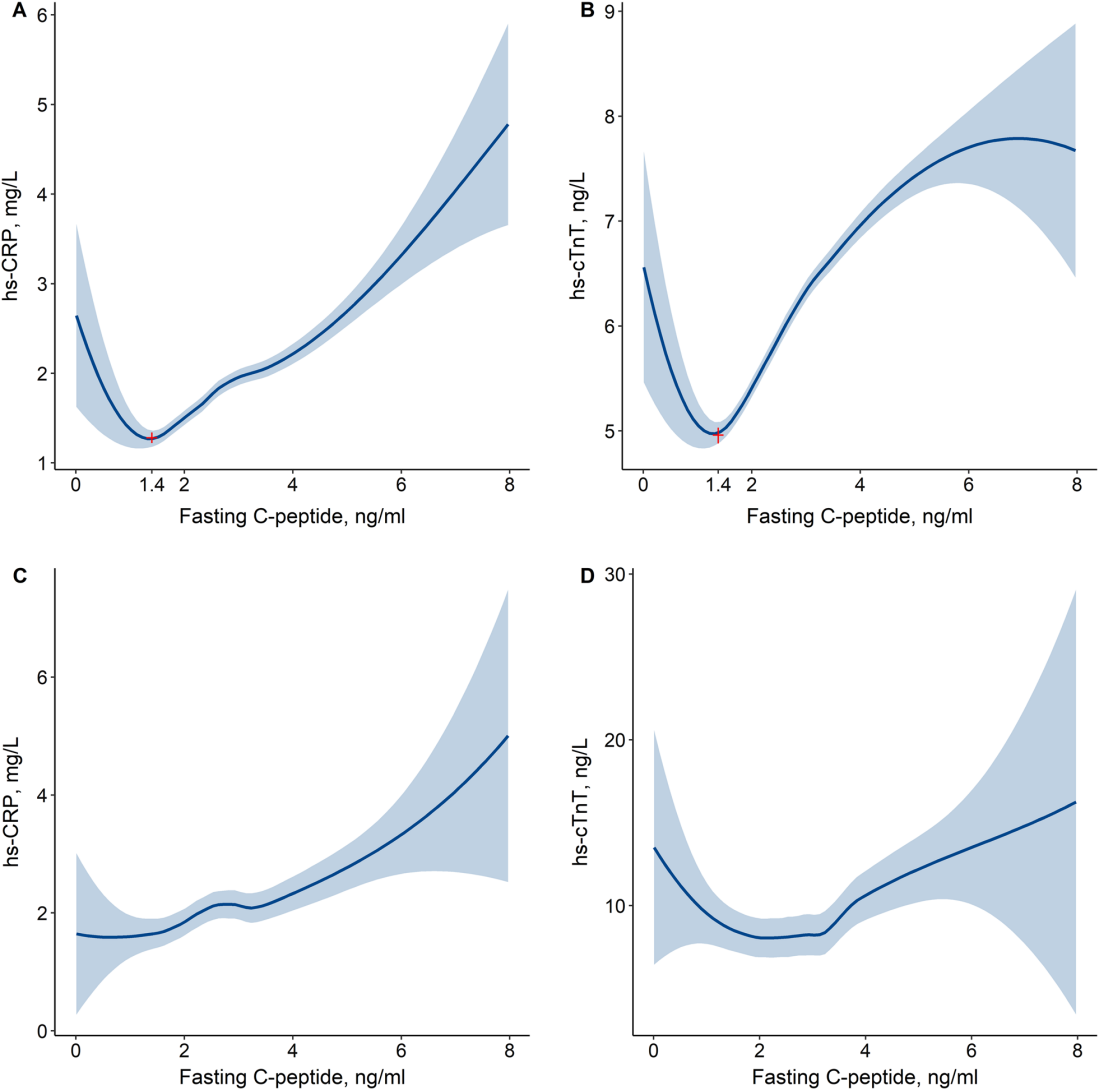
Relationships between fasting C-peptide and cardiovascular biomarkers. **A** and **B** for the participants without previous T2DM. **C** and **D** for the patients with previous T2DM. Locally weighted scatterplot smoothing analyses were used. *hs-CRP* high sensitivity C-reactive protein, *hs-cTnT* high-sensitivity cardiac troponin T, *T2DM* type 2 diabetes mellitus

As presented in Fig. 2, LOESS analyses showed that the relationships between C-peptide and cardiovascular biomarkers were nonlinear and looked like a shape of tick sign or “V” character for the participants without previous T2DM. Generally, both hs-CRP and cTnT levels were negatively correlated with fasting C-peptide under a low level of C-peptide and positively correlated with C-peptide under a high level. However, the relationships between C-peptide and biomarkers were changed in the patients with previous T2DM compared to those without previous T2DM. It seems that fasting C-peptide was positively correlated with hs-CRP and not significantly correlated with hs-cTnT in the patients with previous T2DM.

The relationships between C-peptide and biomarkers were unchanged in most groups if the participants without previous T2DM were stratified by glucose metabolism status (Additional file 1: Fig. S1). Nevertheless, the turning point value of fasting C-peptide had a slight deviation among the groups. Furthermore, the relationships between C-peptide and biomarkers in the patients with previous T2DM varied according to the type of antidiabetic medication use and were unlike those in the participants without previous T2DM (Additional file 1: Figs S2 and S3).

### Stratification of serum C-peptide levels for the participants without previous T2DM

As shown in Fig. 2, the value of the turning point was 1.4 ng/mL for fasting C-peptide. Thus, the cutoff value of 1.4 ng/mL for fasting C-peptide was used to stratify the participants without previous T2DM into two groups. As described in Additional file 1: Table S2, 7.9% (n = 3891) participants had a fasting C-peptide level less than 1.4 ng/mL. Compared to the participants with a high fasting C-peptide level, those with a low C-peptide level had a higher proportion of women, a younger age, lower levels of mean hs-CRP and hs-cTnT, and a lower proportion of prediabetes or ND-T2DM (all P < 0.0001).

Multivariate analyses are described in Table 2. Fasting C-peptide had been shown negative relationships with hs-CRP and hs-cTnT if the value of fasting C-peptide was < 1.4 ng/mL and shown positive relationships if its value was ≥ 1.4 ng/mL in Model 1 adjusting for covariates like sex, age, smoking status, BMI, SBP, LDL cholesterol, triglycerides, previous hypertension, and use of lipid-lowering medication. These relationships remained significant in Model 2 adjusting for covariates in Model 1 plus FPG and 2hPG. After further adjusting for HbA1c, HOMA-IR, and its interaction with fasting C-peptide (Model 3), these relationships were unchanged.

**Table 2:** Multivariate regression models of the relationships between fasting C-peptide and biomarkers in the participants without previous T2DM

	Fasting C-peptide < 1.4 ng/mL, per SD (n = 3891)		Fasting C-peptide ≥ 1.4 ng/mL, per SD (n = 45548)	
	βeta (95% CI)	P value	βeta (95% CI)	P value
hs-CRP, per SD				
Model 1	− 0.059 (− 0.091, − 0.028)	0.0002	0.064 (0.053, 0.075)	< 0.0001
Model 2	− 0.056 (− 0.088, − 0.025)	0.0005	0.056 (0.044, 0.067)	< 0.0001
Model 3	− 0.056 (− 0.088, − 0.023)	0.0007	0.063 (0.047, 0.079)	< 0.0001
hs-cTnT, per SD				
Model 1	− 0.056 (− 0.084, − 0.028)	0.0001	0.073 (0.063, 0.084)	< 0.0001
Model 2	− 0.053 (− 0.082, − 0.025)	0.0002	0.062 (0.051, 0.073)	< 0.0001
Model 3	− 0.053 (− 0.081, − 0.025)	0.0003	0.087 (0.071, 0.102)	< 0.0001

Model 1 was adjusted for sex, age, smoking status, history of hypertension, lipid-lowering medication use, BMI, SBP, LDL cholesterol, and triglycerides. Model 2 was adjusted for covariates in Model 1, FPG, and 2hPG. Model 3 was adjusted for covariates in Model 1, HbA1c, HOMA-IR, and the interaction between fasting C-peptide and HOMA-IR. All continuous variables in the models were standardized and those with a skewness distribution were log transformed

*BMI* body mass index, *CI* confidence interval, *FPG* fasting plasma glucose, *HbA1c* hemoglobin A1c, *HOMA-IR* homeostasis model assessment of insulin resistance, *hs-CRP* high sensitivity C-reactive protein, *hs-cTnT* high-sensitivity cardiac troponin T, *LDL cholesterol*, low-density lipoprotein cholesterol, *ND-T2DM* newly diagnosed T2DM, *NGM* normal glucose metabolism, *SBP* systolic blood pressure, *SD* standard deviation, *T2DM* type 2 diabetes mellitus, *2hPG* 2 h plasma glucose

**Fig. 3:**
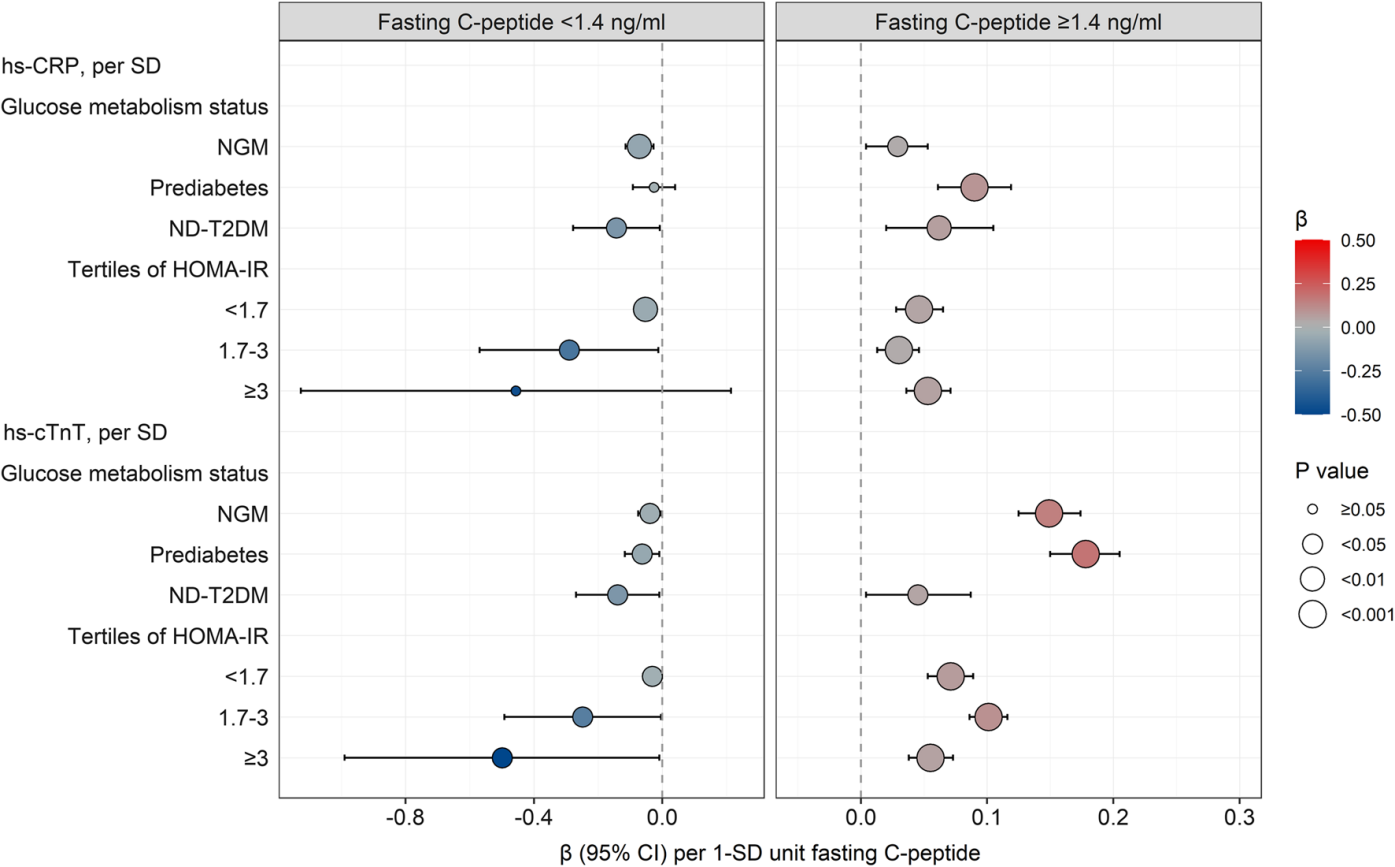
β (95% CI) for relationships of fasting C-peptide with hs-CRP and hs-cTnT in the participants without previous T2DM and stratified by glucose metabolism status or tertiles of HOMA-IR. Covariates included sex, age, smoking status, history of hypertension, lipid-lowering medication use, BMI, SBP, LDL cholesterol, triglycerides, HbA1c, HOMA-IR, and the interaction between fasting C-peptide and HOMA-IR. All continuous variables in the models were standardized and those with a skewness distribution were log transformed. *BMI* body mass index, *CI* confidence interval, *FPG* fasting plasma glucose, *HbA1c* hemoglobin A1c, *HOMA-IR* homeostasis model assessment of insulin resistance, *hs-CRP* high sensitivity C-reactive protein, *hs-cTnT* high-sensitivity cardiac troponin T, *LDL cholesterol* low-density lipoprotein cholesterol, *ND-T2DM* newly diagnosed T2DM, *NGM* normal glucose metabolism, *SBP* systolic blood pressure, *SD* standard deviation, *T2DM* type 2 diabetes mellitus, *2hPG* 2 h plasma glucose

The participants without previous T2DM were further stratified by glucose metabolism status or tertiles of HOMA-IR levels. As presented in Fig. 3 and Additional file 1: Table S3, almost all glucose metabolism subgroups, including NGM, prediabetes, and ND-T2DM, had shown similar relationships between serum C-peptide levels and cardiovascular biomarkers. Furthermore, these relationships were unchanged in each subgroup stratified by tertiles of HOMA-IR levels.

### Serum C-peptide and cardiovascular events

As shown in Additional file 1: Fig. S4 and Table S4, the association between serum C-peptide and the prevalence of cardiovascular events was like the relationships between C-peptide and cardiovascular biomarkers. For the participants without previous T2DM, the prevalence of cardiovascular events was first decreased and then increased with the increasing of baseline C-peptide level. However, for the participants with previous T2DM, the association between C-peptide and cardiovascular events was not significant.

**Fig. 4:**
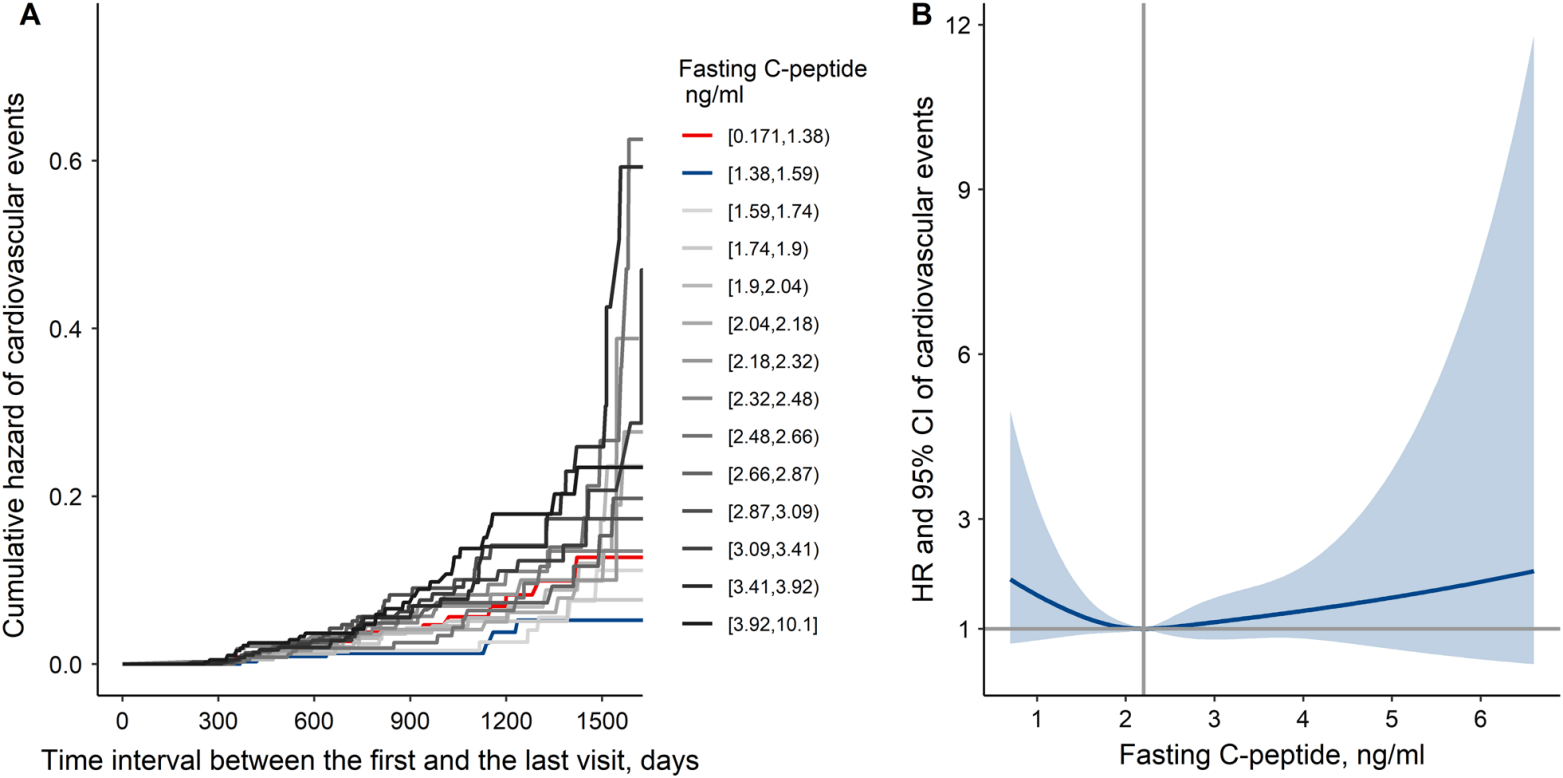
Kaplan–Meier curve and restricted cubic spline for the association between fasting C-peptide and cardiovascular events in the participants without previous T2DM. HRs and 95% CIs were derived from a Cox proportional hazard model adjusting for sex, age, smoking status, use of antihypertensive medication, use of lipid-lowering medication, use of antidiabetic medication, BMI, SBP, LDL cholesterol, triglycerides, HbA1c, HOMA-IR, and the interaction between fasting C-peptide and HOMA-IR. *BMI* body mass index, *CI* confidence interval, *FPG* fasting plasma glucose, *HbA1c* hemoglobin A1c, *HOMA-IR* homeostasis model assessment of insulin resistance, *HR* hazard ratio, *LDL cholesterol* low-density lipoprotein cholesterol, *T2DM* type 2 diabetes mellitus, *2hPG* 2 h plasma glucose

The Kaplan–Meier curve showed that the cumulative hazard of cardiovascular events increased with the increasing of the time interval between the first and the last visit and was higher in those with a lower level or a higher level of fasting C-peptide for the participants without previous T2DM (Fig. 4A). The restricted cubic spline also showed that HRs and 95% CIs of cardiovascular events were also first decreased and then increased with the increasing of baseline C-peptide levels, though these associations became unsignificant using the multivariate Cox regression model (Fig. 4B). These results were unchanged even if the participants who took antidiabetic medication during the visits were excluded (Additional file 1: Fig. S5).

## Discussion

In this retrospective cohort study, we found that the associations of fasting C-peptide with cardiovascular biomarkers and events were different between the participants without previous T2DM and those having previous T2DM. In participants without previous T2DM, these associations were nonlinear, looked like a shape of tick sign or “V” character. These associations were nearly unchanged even if glucose metabolism status, HOMA-IR levels, and the interaction between C-peptide and HOMA-IR were considered. In participants with previous T2DM, however, the associations between serum C-peptide levels and cardiovascular biomarkers as well as events seemed to be unsignificant.

Both hs-CRP and hs-cTnT were widely used to assess cardiovascular risk, identifying early cardiac injury and inflammation in a general population and diabetic patients. Their associations with traditional blood glucose-related markers, rather than C-peptide, were widely reported. Although C-peptide was recognized as an important marker insulin secretion in the literature, its bioactive effects on cardiovascular disease and complications expose attracting attention of researchers in recent years [20, 21].

Generally, the role of C-peptide is controversial and still unclear. In T1DM patients, C-peptide may attenuate hyperglycemia-induced oxidative stress and endothelial dysfunction. It seems that the absence of C-peptide may effectuate increased cardiovascular risk in T1DM patients. However, in nondiabetic individuals, several studies reported a positive linear association between C-peptide and arterial stiffness markers [7, 22]. A higher level of C-peptide had been reported to be correlated with higher risks of subclinical myocardial injury and all-cause and cardiovascular-related mortality [8–10, 23]. Recently, several adverse effects of C-peptide at a low level had been reported [24, 25]. For instance, C-peptide might exert an insulin-independent effect on bone mass and a lower level of C-peptide meant a higher risk of osteoporosis in postmenopausal women without diabetes [25].

Our study observed that the association between C-peptide and cardiovascular risk looked like a shape of tick sign or “V” character in the nondiabetic adults and those with newly diagnosed T2DM. Similarly, a U-shaped association of C-peptide with all-cause and cardiovascular disease mortality had been reported in a non-alcoholic fatty liver disease population [24]. Based on the reports of the beneficial roles of C-peptide against hyperglycemia-induced inflammation and vasculopathy [2], the proinflammatory and arteriosclerosis effects of high levels of C-peptide in insulin resistance and early T2DM [11, 26, 27], and the main findings of the present study, we think that the effect of C-peptide on cardiovascular risk may be bidirectional, negative under a lower level of C-peptide and positive under a higher level, in nondiabetic adults and patients with early diagnosed T2DM. Jannette M. Dufour et al. [1] considered an explanation of receptor saturation for C-peptide. The normal concentration of C-peptide may provide a protective effect, while delivery of excess C-peptide may provide no additional benefit and even provide a harmful effect. The receptor saturation level of C-peptide was reported to be 3 ng/mL and the normal physiological concentration of C-peptide was between 0.5 and 2 ng/mL during fasting [1, 28]. Comparatively, the mean level of fasting C-peptide was 2.5 ng/mL and the turning point was 1.4 ng/mL for the participants without a history of T2DM in our study. The threshold value was reported to be 1.23 ng/mL in the non-alcoholic fatty liver disease population [24]. It means the normal physiological concentration of fasting C-peptide and its receptor saturation level need to be estimated in future studies.

The effect of C-peptide in type 2 diabetic patients is controversial and more complicated. Serum C-peptide levels were reported to be inversely associated with the prevalence of diabetic retinopathy and cardiovascular autonomic neuropathy in patients with T2DM [29, 30]. Low C-peptide level was associated with increased cardiovascular risk in T2DM [31], even if glucose variation or severe hypoglycemia was considered [32]. Nevertheless, the positive association between C-peptide and cardiometabolic biomarkers in patients with T2DM had also been reported [33]. We observed that the association of C-peptide with cardiovascular risk was different between the participants without previous T2DM and those with previous T2DM. We did not observe the bidirectional association in the latter. We suppose that the disease duration and progresses of T2DM may influence C-peptide levels and its association with cardiovascular risk. In the present study, serum C-peptide levels varied depending on the stage of the T2DM disease. The mean level of fasting C-peptide was 2.31 ng/mL in NGM participants, 2.64 ng/mL in participants with prediabetes, 2.97 ng/mL in participants with ND-T2DM, and 2.68 ng/mL in participants with previous T2DM. Furthermore, the influence of diabetes treatment on C-peptide levels should also be considered. We observed a variety of relationship between levels of C-peptide and cardiovascular biomarkers in different antidiabetic medications. It was reported that C-peptide levels were significantly decreased in patients with the sodium-glucose cotransporter 2 inhibitor tofogliflozin treatment but not conventional treatment [34].

The study has several limitations. First, although the participants who visited the hospital at least twice were considered, their numbers were not enough to observe the significant association between C-peptide and hazard of cardiovascular events in the multivariate Cox regression model. Thus, more large-scale cohort studies are needed. Second, although the turning point of 1.4 ng/mL for fasting C-peptide was used, our data showed that this value might be not suitable for all groups stratified by glucose metabolism status. Meanwhile, the turning point for C-peptide seemed to be 2.2 ng/mL in the restricted cubic spline. Thus, the turning point used in our study could not be recommended before further verification. Third, although antidiabetic medications were considered, we did not explore the influence of different medication use on C-peptide levels and cardiovascular risk. The confounding effects of diabetes treatment should be further evaluated for the patients with previous T2DM.

## Conclusions

The present study found that the associations of C-peptide with cardiovascular biomarkers and events were different between the participants without previous T2DM and those having previous T2DM. In the former, these associations were negative a low level of C-peptide and positive a high level. In the latter, these associations seemed to be unsignificant. These findings suggest that the effect of C-peptide on cardiovascular risk may be bidirectional in the nondiabetic adults and the patients with newly diagnosed T2DM. We suppose that C-peptide may play a benefit role at a low level and a harmful role at a high level. Nevertheless, the role of C-peptide in human physiology may be complex. Further researches are needed to investigate the role of C-peptide, the mechanism behind the role, and the difference in this role among nondiabetic adults and patients with T2DM.

## Supplementary Information

**Additional file 1: Table S1.** Baseline characteristics and cardiovascular events in the participants who visited the hospital at least twice. **Table S2.** Differences in demographic characteristics among the groups stratified by levels of fasting C-peptide for the participants without previous T2DM. **Table S3.** Sensitivity analyses of the associations stratified by glucose metabolism status and tertiles of HOMA-IR for the participants without previous T2DM. **Table S4.** Prevalence of cardiovascular events in the participants stratified by quantiles of fasting C-peptide levels. **Fig. S1.** Relationships between fasting C-peptide and cardiovascular biomarkers among the different glucose metabolism status subgroups for the participants without previous T2DM. hs-CRP, high sensitivity C-reactive protein; hs-cTnT, high-sensitivity cardiac troponin T; ND-T2DM, newly diagnosed T2DM; NGM, normal glucose metabolism; T2DM, type 2 diabetes mellitus. **Fig. S2.** Relationships between fasting C-peptide and hs-CRP levels for the participants with previous T2DM stratified by use of antidiabetic medication. DDD-4, dipeptidyl peptidase-4; GLP-1, glucagon-like peptide-1; hs-CRP, high sensitivity C-reactive protein; SGLT2, sodium-glucose cotransporter 2; T2DM, type 2 diabetes mellitus. **Fig. S3.** Relationships between fasting C-peptide and hs-cTnT levels for the participants with previous T2DM stratified by use of antidiabetic medication. DDD-4, dipeptidyl peptidase-4; GLP-1, glucagon-like peptide-1; hs-cTnT, high-sensitivity cardiac troponin T; SGLT2, sodium-glucose cotransporter 2; T2DM, type 2 diabetes mellitus. **Fig. S4.** Prevalence of cardiovascular events in the participants stratified by quantiles of fasting C-peptide levels. (A) for the participants without previous T2DM. (B) for the patients with previous T2DM. **Fig. S5.** Kaplan-Meier curve and restricted cubic spline for the association between fasting C-peptide and cardiovascular events in the participants who did not have a history of T2DM and did not take antidiabetic medication during the visits.

## Data Availability

Some or all datasets generated during and/or analyzed during the current study are not publicly available but are available from the corresponding author on reasonable request.

## Abbreviations

BMI: Body mass index
CI: Confidence interval
DDD-4: Dipeptidyl peptidase-4
DM: Diabetes mellitus
FPG: Fasting plasma glucose
GLP-1: Glucagon-like peptide-1
HbA1c: Hemoglobin A1c
HOMA-IR: Homeostasis model assessment of insulin resistance
HR: Hazard ratio
hs-CRP: High sensitivity C-reactive protein
hs-cTnT: High-sensitivity cardiac troponin T
LDL cholesterol: Low-density lipoprotein cholesterol
ND-T2DM: Newly diagnosed T2DM
NGM: Normal glucose metabolism
OGTT: Oral glucose tolerance test
SBP: Systolic blood pressure
SD: Standard deviation
SGLT2: Sodium-glucose cotransporter 2
T1DM: Type 1 diabetes mellitus
T2DM: Type 2 diabetes mellitus
2hPG: 2 H plasma glucose
