# Supplementary material for: The bidirectional association of C-peptide with cardiovascular risk in nondiabetic adults and patients with newly diagnosed type 2 diabetes mellitus: a retrospective cohort study
Source: Cardiovasc Diabetol. 2022 Oct 3;21:201. doi: 10.1186/s12933-022-01636-z (PMC9531486; doi:10.1186/s12933-022-01636-z)

Supplemental Material

Title: The bidirectional association of C-peptide with cardiovascular risk in nondiabetic adults and patients with newly diagnosed type 2 diabetes mellitus: a retrospective cohort study

Supplemental Table 1. Baseline characteristics and cardiovascular events in the participants who visited the hospital at least twice

|  | Participants without previous T2DM | Patients with previous T2DM |
| --- | --- | --- |
| Number | 6002 | 725 |
| Age, year | 50.0 ± 9.00 | 55.5 ± 8.43 |
| Sex |  |  |
| Men | 4292 (71.5) | 637 (87.9) |
| Women | 1710 (28.5) | 88 (12.1) |
| Current smoking | 1224 (20.4) | 197 (27.2) |
| Previous hypertension |  |  |
| No | 4614 (76.9) | 335 (46.2) |
| Yes | 1388 (23.1) | 390 (53.8) |
| Lipid-lowering medication use |  |  |
| No | 5181 (86.3) | 489 (67.4) |
| Yes | 821 (13.7) | 236 (32.6) |
| BMI, kg/m^2^ | 25.2 ± 3.30 | 26.7 ± 3.64 |
| SBP, mmHg | 123 ± 18.3 | 130 ± 18.4 |
| LDL cholesterol, mmol/L | 3.11 ± 0.84 | 2.81 ± 0.94 |
| Triglycerides, mmol/L | 1.83 ± 1.49 | 2.19 ± 1.63 |
| FPG, mmol/L | 5.36 ± 0.99 | 7.59 ± 2.21 |
| Fasting insulin, mU/L | 10.8 ± 6.84 | 14.9 ± 42.0 |
| Fasting C-peptide, ng/ml | 2.48 ± 0.93 | 2.70 ± 1.15 |
| 2hPG, mmol/L | 7.97 ± 2.38 | 11.5 ± 3.29 |
| HbA1c, % | 5.6 ± 0.60 | 7.0 ± 1.18 |
| HOMA-IR | 2.65 ± 1.96 | 5.03 ± 12.7 |
| hs-CRP, mg/L | 1.70 ± 4.55 | 1.78 ± 3.54 |
| hs-cTnT, ng/L | 5.58 ± 3.47 | 7.96 ± 6.58 |
| Medication use during the visit |  |  |
| antihypertensive | 1716 (28.6) | 417 (57.5) |
| antidiabetic | 291 (4.85) | 725 (100) |
| lipid-lowering | 1242 (20.7) | 257 (35.4) |
| Cardiovascular events | 300 (5.0) | 98 (13.5) |

Supplemental Table 2. Differences in demographic characteristics among the groups stratified by levels of fasting C-peptide for the participants without previous T2DM

|  | Fasting C-peptide, ng/ml | | |
| --- | --- | --- | --- |
|  | <1.4 | ≥1.4 | P value |
| Number | 3891 | 45548 |  |
| Age, year | 48.2 ± 10.85 | 50.2 ± 9.69 | <0.0001 |
| Sex |  |  | <0.0001 |
| Men | 1677 (43.1) | 30862 (67.8) |  |
| Women | 2214 (56.9) | 14686 (32.2) |  |
| Current Smoking | 410 (10.5) | 9828 (21.6) | <0.0001 |
| History of hypertension |  |  | <0.0001 |
| No | 3632 (93.3) | 34538 (75.8) |  |
| Yes | 259 (6.7) | 11010 (24.2) |  |
| Lipid-lowering medication use |  |  | <0.0001 |
| No | 3650 (93.8) | 39108 (85.9) |  |
| Yes | 241 (6.2) | 6440 (14.1) |  |
| BMI, kg/m^2^ | 21.5 ± 2.60 | 25.3 ± 3.38 | <0.0001 |
| SBP, mmHg | 113 ± 17.1 | 122 ± 18.4 | <0.0001 |
| LDL cholesterol, mmol/L | 3.03 ± 0.83 | 3.18 ± 0.88 | <0.0001 |
| Triglycerides, mmol/L | 0.96 ± 0.53 | 1.87 ± 1.49 | <0.0001 |
| FPG, mmol/L | 4.96 ± 0.81 | 5.50 ± 1.04 | <0.0001 |
| Fasting insulin, mU/L | 3.99 ± 2.78 | 11.3 ± 6.91 | <0.0001 |
| Fasting C-peptide, ng/ml | 1.19 ± 0.17 | 2.62 ± 0.93 | <0.0001 |
| 2hPG, mmol/L | 7.41 ± 2.16 | 8.15 ± 2.40 | <0.0001 |
| HbA1c, % | 5.5 ± 0.64 | 5.7 ± 0.63 | <0.0001 |
| HOMA-IR | 0.89 ± 0.68 | 2.85 ± 2.14 | <0.0001 |
| hs-CRP, mg/L | 1.24 ± 3.58 | 1.73 ± 4.31 | <0.0001 |
| hs-cTnT, ng/L | 4.93 ± 2.84 | 5.93 ± 4.95 | <0.0001 |
| Glucose metabolism status |  |  | <0.0001 |
| NGM | 2606 (67.0) | 22857 (50.2) |  |
| Prediabetes | 1050 (27.0) | 17401 (38.2) |  |
| ND-T2DM | 235 (6.0) | 5290 (11.6) |  |

BMI, body mass index; FPG, fasting plasma glucose; HbA1c, hemoglobin A1c; HOMA-IR, homeostasis model assessment of insulin resistance; hs-CRP, high sensitivity C-reactive protein; hs-cTnT, high-sensitivity cardiac troponin T; LDL cholesterol, low-density lipoprotein cholesterol; ND-T2DM, newly diagnosed T2DM; NGM, normal glucose metabolism; SBP, systolic blood pressure; T2DM, type 2 diabetes mellitus; 2hPG, 2 h plasma glucose.

Supplemental Table 3. Sensitivity analyses of the associations stratified by glucose metabolism status and tertiles of HOMA-IR for the participants without previous T2DM

|  | Fasting C-peptide <1.4 ng/ml, per SD | | |  | Fasting C-peptide ≥1.4 ng/ml, per SD | | |
| --- | --- | --- | --- | --- | --- | --- | --- |
|  | Number | βeta (95% CI) | P value |  | Number | βeta (95% CI) | P value |
| **hs-CRP, per SD** |  |  |  |  |  |  |  |
| Glucose metabolism status | |  |  |  |  |  |  |
| NGM | 2606 | -0.071 (-0.114, -0.027) | 0.0015 |  | 22857 | 0.029 (0.004, 0.053) | 0.0242 |
| Prediabetes | 1050 | -0.026 (-0.092, 0.040) | 0.4403 |  | 17401 | 0.090 (0.061, 0.119) | <0.0001 |
| ND-T2DM | 235 | -0.143 (-0.278, -0.008) | 0.0376 |  | 5290 | 0.062 (0.020, 0.105) | 0.0040 |
| Tertiles of HOMA-IR | |  |  |  |  |  |  |
| <1.7 | 3794 | -0.052 (-0.085, -0.020) | 0.0016 |  | 13162 | 0.046 (0.028, 0.065) | <0.0001 |
| 1.7-3 | 77 | -0.290 (-0.569, -0.012) | 0.0414 |  | 17521 | 0.030 (0.013, 0.046) | 0.0004 |
| ≥3 | 20 | -0.456 (-1.126, 0.214) | 0.1680 |  | 14865 | 0.053 (0.036, 0.071) | <0.0001 |
| **hs-cTnT, per SD** |  |  |  |  |  |  |  |
| Glucose metabolism status | |  |  |  |  |  |  |
| NGM | 2606 | -0.039 (-0.075, -0.003) | 0.0348 |  | 22857 | 0.149 (0.125, 0.174) | <0.0001 |
| Prediabetes | 1050 | -0.063 (-0.117, -0.009) | 0.0218 |  | 17401 | 0.178 (0.150, 0.205) | <0.0001 |
| ND-T2DM | 235 | -0.139 (-0.269, -0.009) | 0.0363 |  | 5290 | 0.045 (0.004, 0.087) | 0.0323 |
| Tertiles of HOMA-IR | |  |  |  |  |  |  |
| <1.7 | 3794 | -0.031 (-0.060, -0.001) | 0.0402 |  | 13162 | 0.071 (0.053, 0.089) | <0.0001 |
| 1.7-3 | 77 | -0.248 (-0.492, -0.004) | 0.0468 |  | 17521 | 0.101 (0.086, 0.116) | <0.0001 |
| ≥3 | 20 | -0.499 (-0.990, -0.009) | 0.0465 |  | 14865 | 0.055 (0.038, 0.073) | <0.0001 |

Covariates included sex, age, smoking status, history of hypertension, lipid-lowering medication use, BMI, SBP, LDL cholesterol, triglycerides, HbA1c, HOMA-IR, and the interaction between fasting C-peptide and HOMA-IR. All continuous variables in the models were standardized and those with a skewness distribution were log transformed.

BMI, body mass index; CI, confidence interval; FPG, fasting plasma glucose; HbA1c, hemoglobin A1c; HOMA-IR, homeostasis model assessment of insulin resistance; hs-CRP, high sensitivity C-reactive protein; hs-cTnT, high-sensitivity cardiac troponin T; LDL cholesterol, low-density lipoprotein cholesterol; ND-T2DM, newly diagnosed T2DM; NGM, normal glucose metabolism; SBP, systolic blood pressure; SD, standard deviation; T2DM, type 2 diabetes mellitus; 2hPG, 2 h plasma glucose.

Supplemental Table 4. Prevalence of cardiovascular events in the participants stratified by quantiles of fasting C-peptide levels.

|  | Participants without previous T2DM | | |  | Patients with previous T2DM | | |
| --- | --- | --- | --- | --- | --- | --- | --- |
|  | Number | Events | Rate, %  (95% CI) |  | Number | Events | Rate, %  (95% CI) |
| Total | 6002 | 300 | 5.00 (4.45, 5.55) |  | 725 | 98 | 13.5 (11.0, 16.0) |
| Stratification of fasting C-peptide | | |  |  |  |  |  |
| Level 1 | 415 | 18 | 4.34 (2.38, 6.30) |  | 52 | 7 | 13.5 (4.18, 22.7) |
| Level 2 | 432 | 8 | 1.85 (0.58, 3.12) |  | 52 | 10 | 19.2 (8.52, 29.9) |
| Level 3 | 422 | 11 | 2.61 (1.09, 4.13) |  | 52 | 6 | 11.5 (2.85, 20.2) |
| Level 4 | 428 | 13 | 3.04 (1.41, 4.66) |  | 51 | 6 | 11.8 (2.92, 20.6) |
| Level 5 | 432 | 16 | 3.70 (1.92, 5.48) |  | 52 | 6 | 11.5 (2.85, 20.2) |
| Level 6 | 440 | 19 | 4.32 (2.42, 6.22) |  | 52 | 9 | 17.3 (7.03, 27.6) |
| Level 7 | 410 | 19 | 4.63 (2.60, 6.67) |  | 49 | 3 | 6.12 (-0.59, 12.8) |
| Level 8 | 430 | 21 | 4.88 (2.85, 6.92) |  | 53 | 9 | 17.0 (6.87, 27.1) |
| Level 9 | 443 | 22 | 4.97 (2.94, 6.99) |  | 53 | 8 | 15.1 (5.46, 24.7) |
| Level 10 | 429 | 22 | 5.13 (3.04, 7.22) |  | 48 | 2 | 4.17 (-1.49, 9.82) |
| Level 11 | 428 | 27 | 6.31 (4.01, 8.61) |  | 52 | 8 | 15.4 (5.58, 25.2) |
| Level 12 | 428 | 32 | 7.48 (4.98, 9.97) |  | 55 | 4 | 7.27 (0.41, 14.1) |
| Level 13 | 436 | 36 | 8.26 (5.67, 10.8) |  | 52 | 8 | 15.4 (5.58, 25.2) |
| Level 14 | 429 | 36 | 8.39 (5.77, 11.0) |  | 52 | 12 | 23.1 (11.6, 34.5) |

The participants were stratified into fourteen equal subgroups for those without previous T2DM and the patients with previous T2DM, respectively, according to their own C-peptide levels.

CI, confidence interval; T2DM, type 2 diabetes mellitus.

Supplemental Figure 1. Relationships between fasting C-peptide and cardiovascular biomarkers among the different glucose metabolism status subgroups for the participants without previous T2DM. hs-CRP, high sensitivity C-reactive protein; hs-cTnT, high-sensitivity cardiac troponin T; ND-T2DM, newly diagnosed T2DM; NGM, normal glucose metabolism; T2DM, type 2 diabetes mellitus.


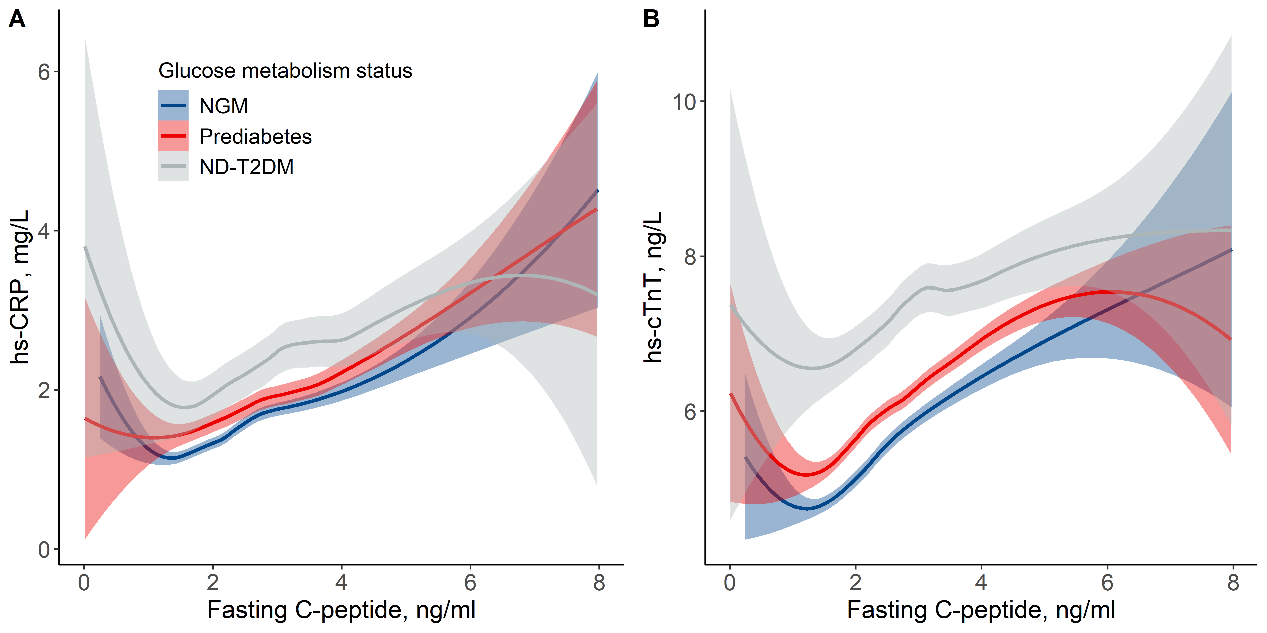


Supplemental Figure 2. Relationships between fasting C-peptide and hs-CRP levels for the participants with previous T2DM stratified by use of antidiabetic medication. DDD-4, dipeptidyl peptidase-4; GLP-1, glucagon-like peptide-1; hs-CRP, high sensitivity C-reactive protein; SGLT2, sodium-glucose cotransporter 2; T2DM, type 2 diabetes mellitus.


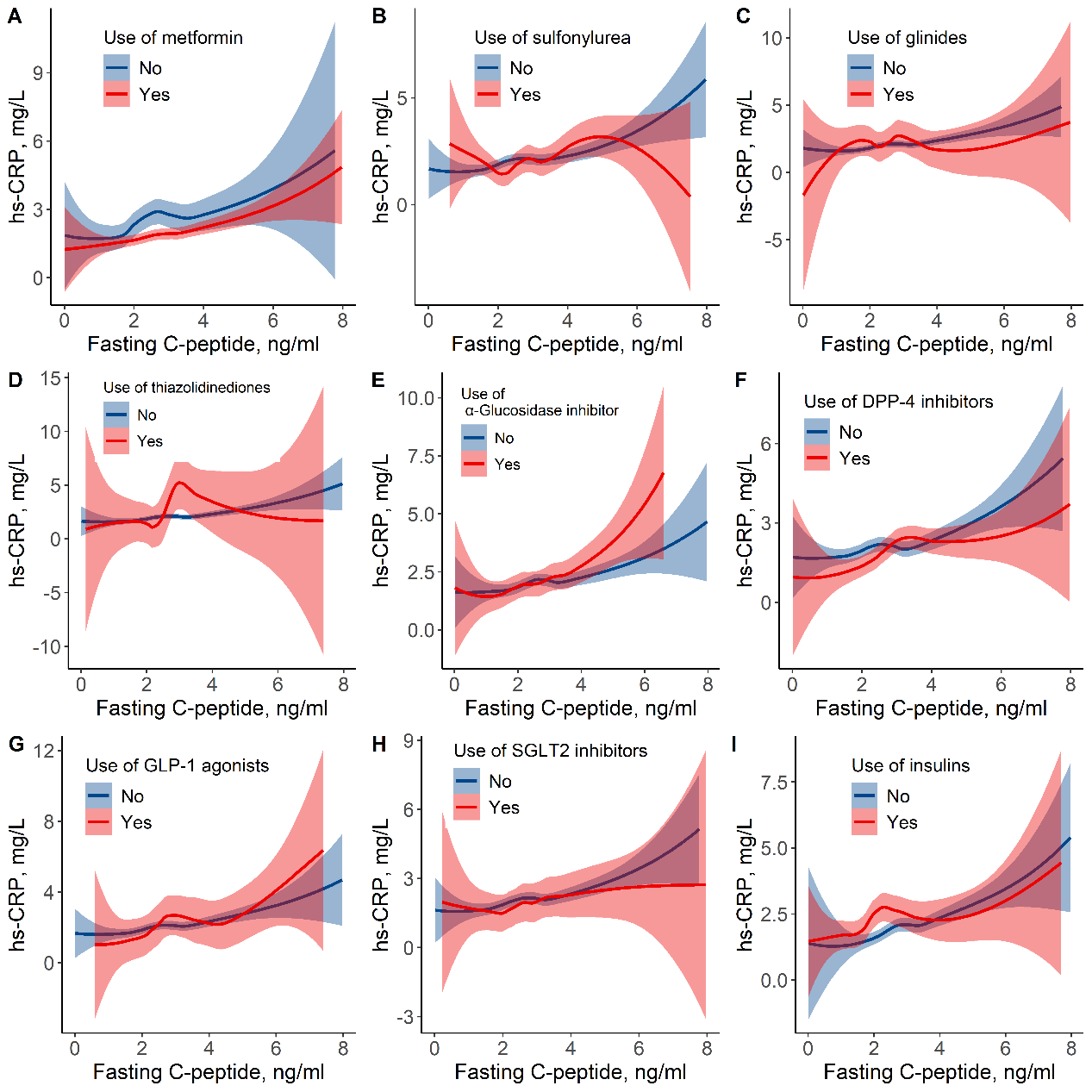


Supplemental Figure 3. Relationships between fasting C-peptide and hs-cTnT levels for the participants with previous T2DM stratified by use of antidiabetic medication. DDD-4, dipeptidyl peptidase-4; GLP-1, glucagon-like peptide-1; hs-cTnT, high-sensitivity cardiac troponin T; SGLT2, sodium-glucose cotransporter 2; T2DM, type 2 diabetes mellitus.


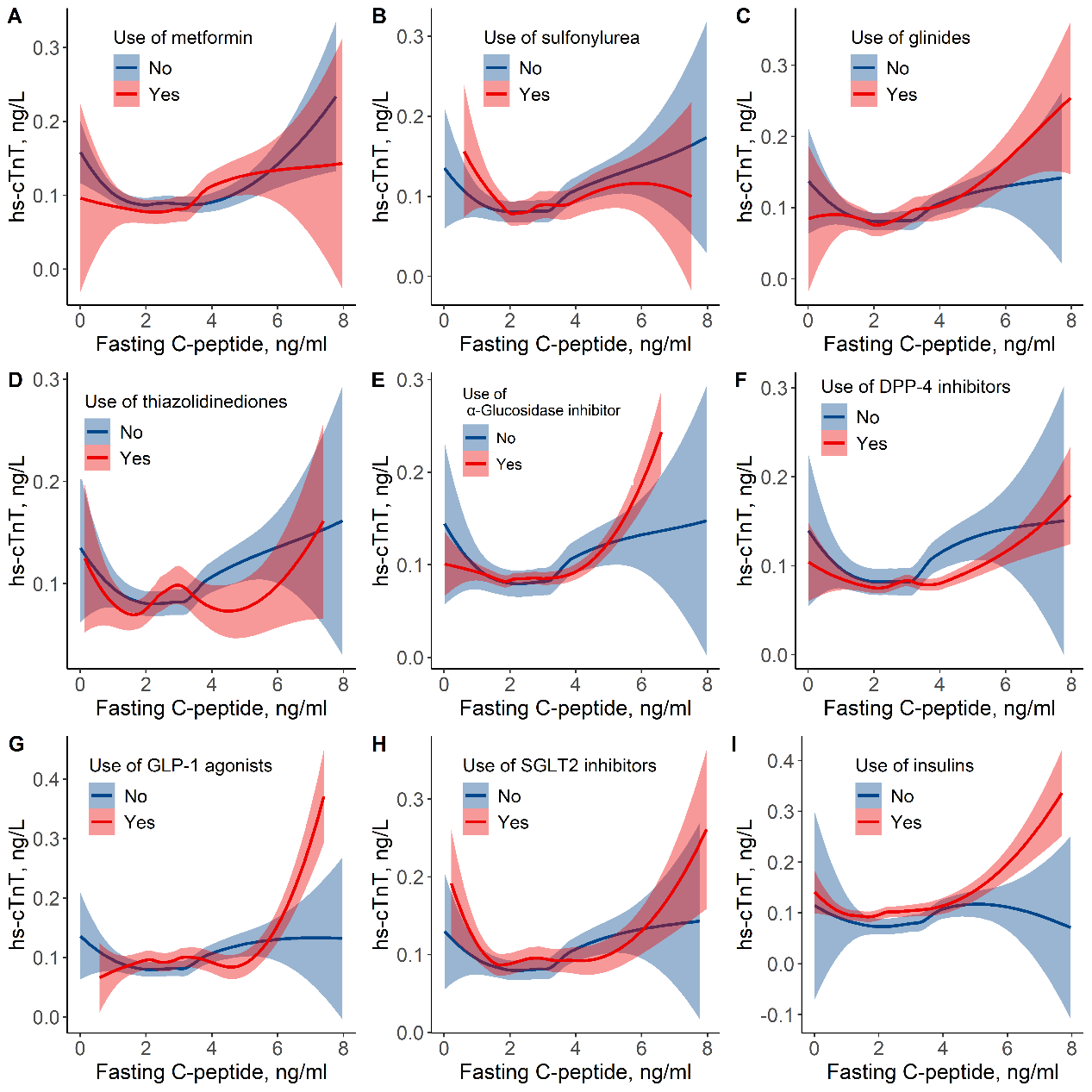


Supplemental Figure 4. Prevalence of cardiovascular events in the participants stratified by quantiles of fasting C-peptide levels. (A) for the participants without previous T2DM. (B) for the patients with previous T2DM.

T2DM, type 2 diabetes mellitus.


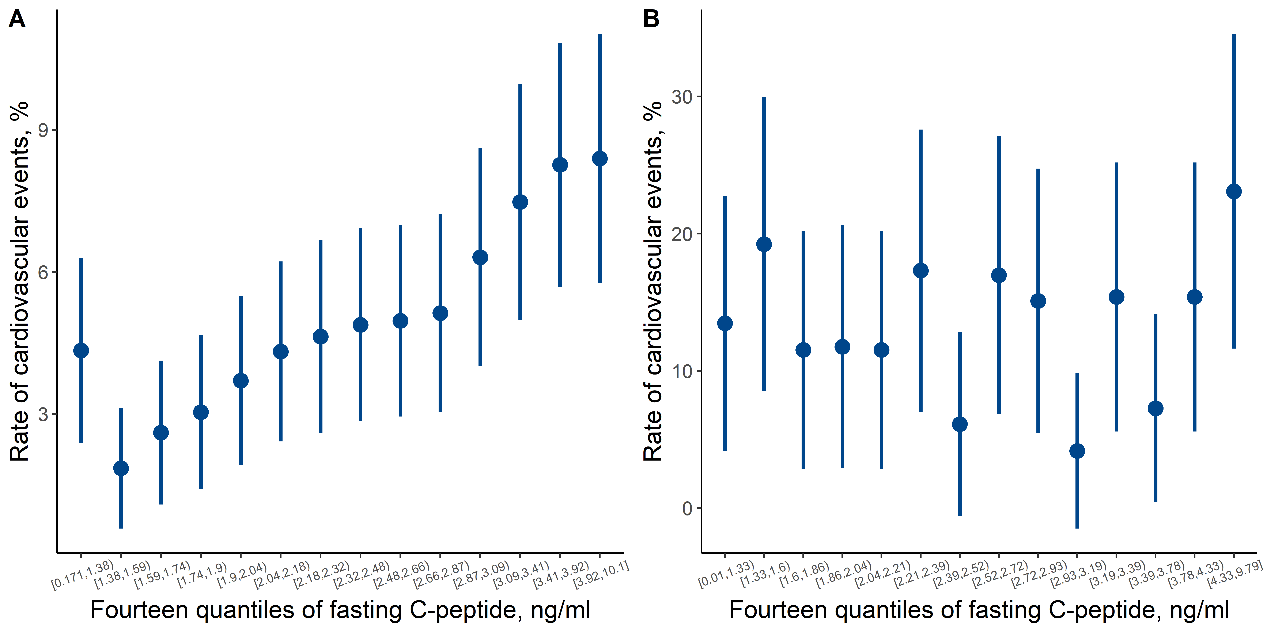


Supplemental Figure 5. Kaplan-Meier curve and restricted cubic spline for the association between fasting C-peptide and cardiovascular events in the participants who did not have a history of T2DM and did not take antidiabetic medication during the visits.

HRs and 95% CIs were derived from a Cox proportional hazard model adjusting for sex, age, smoking status, use of antihypertensive medication, use of lipid-lowering medication, use of antidiabetic medication, BMI, SBP, LDL cholesterol, triglycerides, HbA1c, HOMA-IR, and the interaction between fasting C-peptide and HOMA-IR.

BMI, body mass index; CI, confidence interval; FPG, fasting plasma glucose; HbA1c, hemoglobin A1c; HOMA-IR, homeostasis model assessment of insulin resistance; HR, hazard ratio; LDL cholesterol, low-density lipoprotein cholesterol; T2DM, type 2 diabetes mellitus; 2hPG, 2 h plasma glucose.


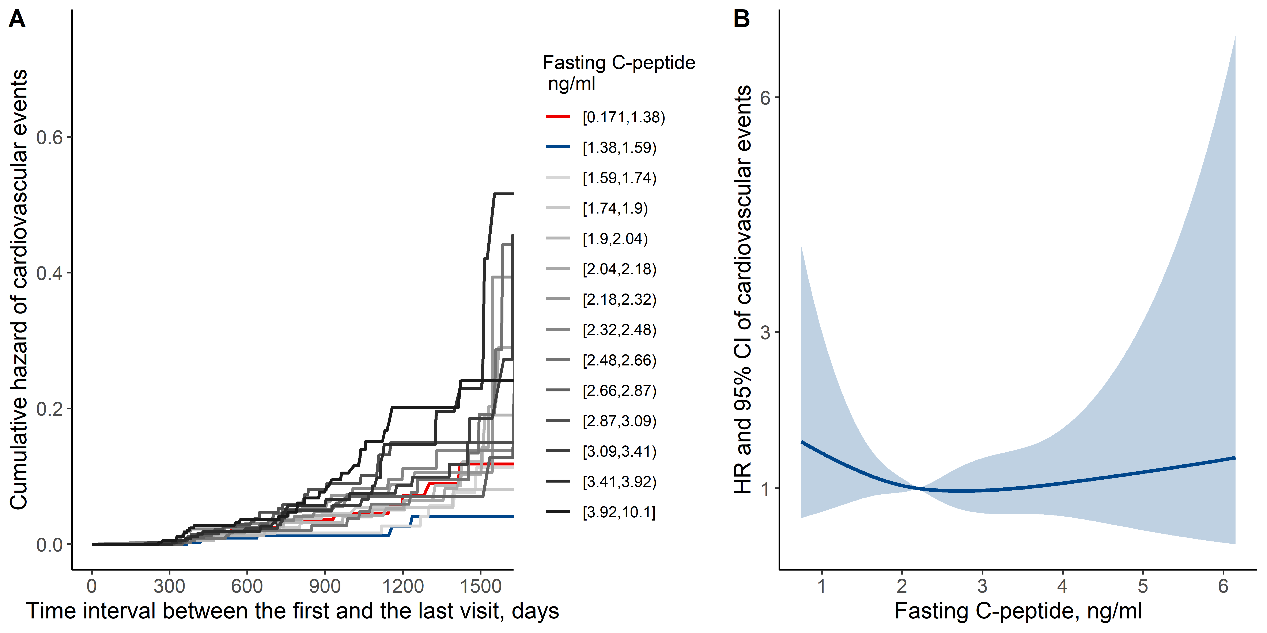

Supplement: Supplementary file 1 — Additional file 1: Table S1. Baseline characteristics and cardiovascular events in the participants who visited the hospital at least twice. Table S2. Differences in demographic characteristics among the groups stratified by levels of fasting C-peptide for the participants without previous T2DM. Table S3. Sensitivity analyses of the associations stratified by glucose metabolism status and tertiles of HOMA-IR for the participants without previous T2DM. Table S4. Prevalence of cardiovascular events in the participants stratified by quantiles of fasting C-peptide levels. Fig. S1. Relationships between fasting C-peptide and cardiovascular biomarkers among the different glucose metabolism status subgroups for the participants without previous T2DM. hs-CRP, high sensitivity C-reactive protein; hs-cTnT, high-sensitivity cardiac troponin T; ND-T2DM, newly diagnosed T2DM; NGM, normal glucose metabolism; T2DM, type 2 diabetes mellitus. Fig. S2. Relationships between fasting C-peptide and hs-CRP levels for the participants with previous T2DM stratified by use of antidiabetic medication. DDD-4, dipeptidyl peptidase-4; GLP-1, glucagon-like peptide-1; hs-CRP, high sensitivity C-reactive protein; SGLT2, sodium-glucose cotransporter 2; T2DM, type 2 diabetes mellitus. Fig. S3. Relationships between fasting C-peptide and hs-cTnT levels for the participants with previous T2DM stratified by use of antidiabetic medication. DDD-4, dipeptidyl peptidase-4; GLP-1, glucagon-like peptide-1; hs-cTnT, high-sensitivity cardiac troponin T; SGLT2, sodium-glucose cotransporter 2; T2DM, type 2 diabetes mellitus. Fig. S4. Prevalence of cardiovascular events in the participants stratified by quantiles of fasting C-peptide levels. (A) for the participants without previous T2DM. (B) for the patients with previous T2DM. Fig. S5. Kaplan-Meier curve and restricted cubic spline for the association between fasting C-peptide and cardiovascular events in the participants who did not have a history of T2DM and [file 12933_2022_1636_MOESM1_ESM.docx]
